# Biomarkers of Exposure to Chemical Contamination in the Commercial Fish Species *Lepidopus caudatus* (Euphrasen, 1788): A Particular Focus on Plastic Additives

**DOI:** 10.3389/fphys.2019.00905

**Published:** 2019-07-16

**Authors:** Antonio Salvaggio, Francesco Tiralongo, Evangelia Krasakopoulou, Dimitra Marmara, Ioannis Giovos, Rosalia Crupi, Giuseppina Messina, Bianca Maria Lombardo, Alessandra Marzullo, Roberta Pecoraro, Elena Maria Scalisi, Chiara Copat, Pietro Zuccarello, Margherita Ferrante, Maria Violetta Brundo

**Affiliations:** ^1^Experimental Zooprophylactic Institute of Sicily A. Mirri, Palermo, Italy; ^2^Department of Biological, Geological and Environmental Sciences, University of Catania, Catania, Italy; ^3^Department of Marine Sciences, University of the Aegean, Mytilene, Greece; ^4^iSEA, Environmental Organization for the Preservation of the Aquatic Ecosystems Ochi Av., Thessaloniki, Greece; ^5^Department of Chemical, Biological, Pharmaceutical and Environmental Sciences, University of Messina, Messina, Italy; ^6^Department of Medical, Surgery Sciences and Advanced Technologies, G. F. Ingrassia, University of Catania, Catania, Italy

**Keywords:** fish, phthalates, bisphenol A, heavy metals, biomarkers

## Abstract

In recent years, the Mediterranean Sea has become an accumulation zone for waste generated by the 22 countries bordering its shores. Although the effects of plastic litter on the marine environment and on organisms have recently been studied in other areas, further information is needed for the Mediterranean Sea and, in particular, about plastics additives inputs and interactions with the biota and the trophic network, such as phthalates and bisphenol A. Plastic material production, use and disposal contribute also to the release of heavy metals into the environment, such as mercury (Hg), often used during the production of chlorine, the primary ingredient in PVC, lead (Pb) and cadmium (Cd), which are used as stabilizers in PVC and leach out of products during use and disposal. Our research aims to evaluate phthalates, bisphenol A and heavy metals contamination in *Lepidopus caudatus* (Pisces, Trichiuridae), which could be considered as a potential sentinel species. For the evaluation of toxicological effects, we evaluated the expression of vitellogenin and metallothioneins 1. In all samples analyzed, we have not found microplastics in the gastrointestinal tract but chemical analysis revealed the presence of high content of phthalates, and in particular high quantities of DIDP, DEHP, bis-benzylester phthalate, bis-butyl ester phthalate and mono-N-butyl ester phthalate in different organs. Instead, trace elements detected in tissue revealed a trend of concentrations generally higher in liver and intestine than gill and muscle tissues. Immunohistochemical analysis for anti-metallothionein 1 antibody showed a strong positivity of liver cells, both in females and males. Analysis for the anti-vitellogenin antibody showed in females a strong positivity both in the liver cells and in the gonads, in male specimens was found to be always negative except for a specimen, in which it was highlighted a positivity in some areas of the liver and of the gonad.

## Introduction

Marine pollution is one of the biggest threats on a global scale to the heart health. In the last few decades, bioaccumulation studies performed with a multimarkers approach have been a valuable tool for the investigation of environmental and animal safety (Ferrante et al., 2017). Together with the climate change, plasticizers represent an emerging problem that could influence the human ability to preserve biological diversity in the future (Jambeck et al., 2015). It is estimated that about eight million tons of plastic reach the marine environment each year (Jambeck et al., 2015). The Mediterranean Sea, a crucial hotspot for biodiversity, has been described as one of the areas most affected by marine litter in the world (Cozar et al., 2015; UNEP, 2016). Plastic accounts for 95% of the offshore waste, on the seabed and on the beaches of the Mediterranean Sea, and comes mainly from Turkey and Spain, followed by Italy, Egypt and France (Alessi et al., 2018). Therefore, the dramatic increase in the use of plastic materials in the last decades has led to the dispersion of plasticizers in the marine environment (Zuccarello et al., 2018).

Recently, awareness is growing about how the smaller plastic fragments, the so-called microplastics (fragments less than 5 mm), are harmful and dangerous to the environment and human health (Zuccarello et al., 2019). Their impact on the marine ecosystem is still under investigation, although some important implications are already known, such as the possibility to be ingested by a wide variety of marine organisms and the consequent introduction into trophic cycle (Ashton et al., 2010; Fossi et al., 2012; Seltenrich, 2015) with deleterious effects on health of marine organisms (e.g., abrasions, blockage of the digestive tract, absorption of harmful compounds) (Rios et al., 2007; Teuten et al., 2009; Engler, 2012). Plastic additives, such as phthalates and bisphenol A (BPA), are added to plastics during production processes in order to improve their properties. Since they are not covalently bound but simply mixed with the plastic polymer, they disperse easily in the environment, especially when plastic products are degraded into microplastics (Hermabessiere et al., 2017; Savoca et al., 2018). Also, many heavy metals such as Cd, Pb, Sb and Sn (as organotin) have been used as plastic additives, and inappropriate plastic use, disposal and recycling may lead to their undesirable release (Hahladakis et al., 2018).

In addition, due to their hydrophobic nature and to the large surface/volume ratio, microplastics are can absorb a series of toxic from seawater, bioaccumulative and persistent substances, such as polycyclic aromatic hydrocarbons (PAHs), polychlorinated biphenyls (PCBs), other persistent organic pollutants (POPs) and heavy metals (Teuten et al., 2009; Rochman et al., 2014). Single and combined effects of microplastics and other contaminants were studied in some marine organisms (Davarpanah and Guilhermino, 2015; Karami et al., 2016).

These chemicals have the potential to accumulate in the tissues of marine organisms and cause specific effects, including behavioral changes, changes in metabolic processes and endocrine disruption (Anderson et al., 2016). The endocrine system performs fundamental tasks for the life of an organism and the hormones produced by the endocrine glands have the task of controlling delicate and complex phenomena such as reproduction, growth, development, as well as the metabolism. Endocrine disrupters (EDCs) are able to imitate, compete or stop the synthesis of endogenous hormones (Avio et al., 2015, 2017); this translates into alterations of glands’ function, alteration and reduction of reproduction with consequent low birth rates and potential loss of biodiversity (Gore et al., 2015). Compounds identified as EDCs, such as phthalates, bisphenol A and heavy metals, are a major concern for marine organisms. Low-level exposure of these substances leads to both transient and permanent changes in the endocrine system (Greenwell, 2014; Zuccarello et al., 2018). Phthalates and bisphenol A, showed to have a role in the development of obesity and glucose metabolism disorders (Stojanoska et al., 2017). Exposure to phthalates, particularly to DEHP, causes a decrease in the production of testicular testosterone in rodents, and most of the reproductive toxic effects are suggested to be related to their antiandrogenic potential (Jones et al., 1993; Erkekoglu et al., 2011). These substances were shown to cause both hepatocellular carcinomas and adenomas (Kluwe et al., 1985; Astill et al., 1996) and were suggested to be toxic to kidneys, thyroid and to the neuroendocrine system (Erkekoglu et al., 2012a,b; Liu et al., 2014). BPA was shown to alter mammary gland development and increase the incidence of tumors in Sprague-Dawley rats (Soto et al., 2013), causes development of breast, prostate, and nipple cancers (Keri et al., 2007), alter the development of reproductive organs, the testosterone excretion, and the sperm production (Richter et al., 2007). Heavy metals have also been recognized as likely inducers of testicular damage, although the mechanisms of testicular toxicity exerted by heavy metals are still unclear (De Toni et al., 2017). Several epidemiological studies showed associations between heavy metal exposure and adverse pregnancy outcomes (Motawei et al., 2013; Jameil, 2014; Quansah et al., 2015).

In this perspective, our work aims to evaluate the impact of microplastic, plastic additives and heavy metals contamination in the commercial fish species *Lepidopus caudatus* (Euphrasen, 1788) (Pisces, Trichiuridae), commonly known as silver scabbardfish (spatola in Italian), occurring in temperate waters of all oceans and in the Mediterranean Sea (D’Onghia et al., 2000). We have used the triple approach recently proposed by Fossi et al. (2018), a method that combines a measurement of marine waste and microplastic presence in organisms, the assessment of levels of plastic additives and persistent compounds, and their toxicological effects. This combined analysis allows a more robust assessment of the real impact of contaminants.

The study has been based on the following data: (1) analysis of the gastrointestinal content to evaluate the ingested microplastics; (2) quantitative and qualitative analysis of plastic additives and persistent compounds used as plastics tracers in the tissues of bioindicator organisms; (3) analysis of the toxicological effects of plastic additives ingestion through biomarkers detection and histological analysis.

## Materials and Methods

For this study, 20 specimens (10 males and 10 females) of *L. caudatus* fished in FAO area 37 were analyzed. Fish were captured as part of local commercial fishing with longlines and transported to the laboratory where, in addition to taking samples for chemical and histological analyses, a check of the gastrointestinal contents was performed.

### Determination of Phthalates and Bisphenol A

The extraction, performed by liquid-liquid method, was divided into different phases: aliquots of each sample (specifically muscle, gonad, gill, intestine and liver) were transferred into a glass tube with 2 ml pH 7 of 0.1 M phosphate buffer. After the addition of 3 ml of dichloromethane, the samples have been shaken by vortex and centrifuged for 4 min at 3000 rpm. The organic phase was separated. The extraction was repeated with an additional 2 ml of dichloromethane. The second extract was mixed with the previous one. After evaporation by nitrogen insufflation, the dried extracts were recovered with 50 μl of methanol.

Analysis was performed by Ultra-High-Pressure Liquid Chromatography with Electro-Spray ionization and by Mass Quadrupole Mass Spectrometry. For this purpose, a Waters UHPLC-ESI-TQD Acquity system was used with the Acquity UPLC^®^ HSS C18 1.8 μm – 2.1 × 150mm column and mobile phase consisting in water and methanol (both added at 0.1% with formic acid) for ESI+ analysis and water and methanol (both added at 0.1% with ammonia) for ESI- analysis, in percentages of gradient variables during the race. The mass spectrometry settings were as follows: capillary energy was 3.0 kV, source temperature was 150°C, column temperature was 40°C, desolvation temperature was 500°C, desolvation gas was 1000 L/hr and cone gas was 100 L/hr. The injected sample was 5 microliters.

The reading was performed using the MRM acquisition method, selecting the ionic transitions from the values of m/z (ratio between the mass of the single ion fragment and its electric charge) obtained from the analysis of suitable reference materials and shown in Table 1.

**TABLE 1:** MRM acquisition method.

**Parametri UPLC-MS/MS**	**ESI**	**Sample 1**	**Sample 2**
*DIDP*	+	447 > 141	447 > 288.8
*DINP*	+	419.2 > 127	419.2 > 274
*DEHP*	+	390.7 > 148.7	390.7 > 112.9
*Bis-benzilestere ftalato*	+	346.6 > 180.8	346.6 > 238
*Bis-butilestereftalato*	+	279.2 > 148.8	279.2 > 204,7
*Mono-N-octilestere ftalato*	+	278.9 > 148.8	278.9 > 71
*Mono-N-butilestere ftalato*	+	222.7 > 148.8	222.7 > 204.8
*Mono-metilestere ftalato*	+	180.7 > 148.7	180.7 > 162.7
*BPA*	−	226.8 > 132.8	226.8 > 211.7

Principal Components Analysis (PCA) on the distance matrix was used to identify in which tissue the phthalates presence was highest. Data were standardized before analysis and the results were displayed in a biplot distance (Legendre and Legendre, 2012).

The PCA on the correlation matrix was instead used to evaluate if exist a correlation between the various levels of phthalates. Data were standardized before analysis and the results were visualized in a biplot correlation (Turchetti et al., 2013).

### Trace Elements Analysis

Arsenic (As), cadmium (Cd), cobalt (Co), chromium (Cr), copper (Cu), lead (Pb), mercury (Hg), manganese (Mn), nickel (Ni), vanadium (V), selenium (Se), antimony (Sb) and zinc (Zn), were extracted and quantified in muscle of fish according to the method described in Copat et al. (2018). Briefly, aliquots of 0.5 g of muscle tissue were removed and acid digested in a microwave system (Ethos Touch Control, Milestone S.r.l., Italy) equipped with pressurized vessels, using a digestion solution of 6 ml of 65% nitric acid (HNO_3_) (Carlo Erba) and 2 ml of 30% peroxide hydrogen (H_2_O_2_-Carlo Erba). The quantification of metals was carried on with an ICP-MS Elan-DRC-e (Perkin–Elmer, United States). Analytical blanks, standard and samples were processed with the same acid matrix. Standards for the instrument calibration were prepared with a multi-elements certified reference solution ICP Standard (Merck).

The method detection limits (MDL) estimated with ten blanks was calculated according to the following equation:

MDL = One -tailed student *t*-test (*p* = 0.99%; df = n-1) × Sr

MDL (mg/kg ww) estimated for each trace elements are the following: As 0.013, Cd 0.002, Co 0.008, Cr 0.003, Cu 0.005, Pb 0.001, Hg 0.0025, Mn 0.005, Ni 0.007, V 0.025, Se 0.03, and Zn 0.109.

A laboratory-fortified matrix (LFM) was processed for the quality control with recovery rates between 91.5 and 110%.

Statistical analysis was performed with the software SPSS (version 20.0, Inc., IBM, United States). The normal distribution was verified using the Kolmogorov–Smirnov test. Since several trace element concentrations did not have a normal distribution, the Mann-Whitney non-parametric test was used to compare median concentrations between tissues.

### Histological Analysis

Samples were fixed in 4% formaldehyde (Bio-Optica) in PBS buffered to 0.1 M, pH 7.4 (Sigma Life Science) at room temperature for 36 h. Gills were decalcified, prior to processing, with a decalcifier agent (Biodec R, Bio-Optica) for 3 h at room temperature. Histological examinations were performed according to our standard laboratory procedures (Brundo et al., 2011; Droutsa et al., 2019), and processed with Tissue Processing Center TPC 15 Duo (MEDITE^®^). The sections were stained with Haematoxylin-Eosin (HE) (Bio-Optica) and observed under optical microscope (Leica DM750, Monument, CO, United States) equipped with a digital camera (Leica DFC500, Monument, CO, United States).

### Immunohistochemical Analysis

The immunohistochemical protocol was performed on the liver and gonads sections to detect mouse monoclonal anti-VGT (Abcam, 1:1000) and on liver sections to detect mouse polyclonal anti-MT1 (Abcam, 1:1000); secondary antibody used is FIT-conjugated goat anti-mouse IgG (Sigma-Aldrich, 1:1000). Analysis were performed according to our standard laboratory procedures (Salvaggio et al., 2017; Pecoraro et al., 2018). Slides after mounted with mounting medium containing DAPI (Vectashield, Vector Laboratories), were observed with fluorescent microscope (Olympus Optical U-Ulh, Gilroy, CA, United States).

## Results and Discussion

The stomach content of the collected specimens contained highly digested food items, and in some specimens, we have found the bait fish (*Sardina pilchardus*). We failed to detect the presence of microplastics in the gastrointestinal tract of the fish analyzed, but chemical analysis revealed the presence of a high content of phthalates, and in particular high quantities of DIDP, DEHP, bis-benzylester phthalate, bis-butyl ester phthalate and mono-N-butyl ester phthalate (Table 2).

**TABLE 2:** Results of chemical analysis performed with UPLC-ESI-TQD.

**Analyte**	**Muscle**	**Liver**	**Gonad**	**Gill**	**Intestine**
*DIDP*	9.0	14.5	10.8	<17.9^a^	41.1
*DINP*	3.7^a^	<10.2^a^	<6.8^a^	<17.9^a^	<18.5^a^
*DEHP*	43.3	20.6	30.5	93.6	193.0
*Bis-benzilestere ftalato*	<3.7^a^	<10.2^a^	10.5	<17.9^a^	33.3
*Bis-butilestereftalato*	13.0	<10.2^a^	8.5	29.6	77.8
*Mono-N-octilestere ftalato*	12.4	<10.2^a^	7.3	<17.9^a^	<18.5^a^
*Mono-N-butilestere ftalato*	9.9	<10.2^a^	<6.8^a^	<17.9^a^	54.8
*Mono-metilestere ftalato*	<3.7^a^	<10.2^a^	<6.8^a^	88.2	<18.5^a^
*BPA*	<3.7^a^	24.5	19.6	65.7	<18.5^a^

*^a^ Limit of detection.*

**FIGURE 1:**
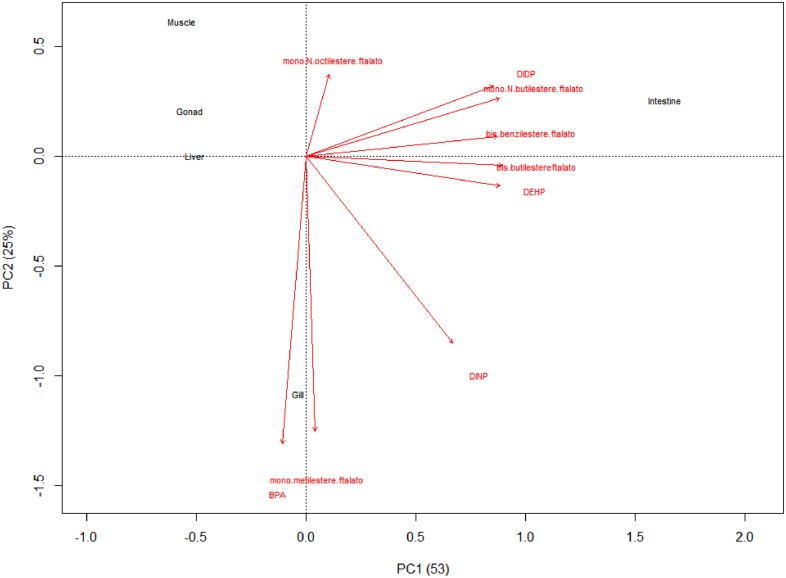
The distance biplot showed that intestine is the most affected to presence of DEHP, DIDP, bis-benzilesterephthalate, bis-butilesterephthalate, mono-N-butilesterephthalate and DINP. Instead, BPA and mono-metilesterephthalates were strongly present in gill. Otherwise, muscle, gonad and liver showed low levels of phthalates and BPA.

The distance biplot (PC1 and PC2 were 53 and 25%, respectively) showed that intestine is the most affected by the presence of DEHP, DIDP, bis-benzilesterephthalate, bis-butilesterephthalate, mono-N-butilesterephthalate and DINP (Figure 1). Instead, BPA and mono-metilesterephthalates were strongly present in gill. Otherwise, muscle, gonad and liver showed low levels of phthalates and BPA (Figure 1).

**FIGURE 2:**
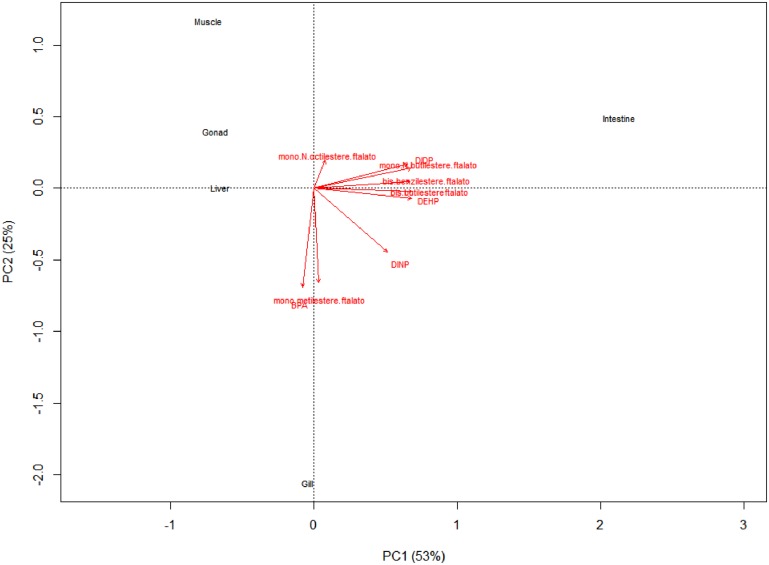
Correlation biplot showed simultaneous presence of DEHP, DIDP, bis-benzilesterephthalate, bis-butilesterephthalate and mono-N-butilesterephthalate while BPA was related to presence of mono-metilesterephthalate. Otherwise, DINP and mono-N-octilesterephthalate were weakly related to others.

Correlation biplot (PC1 and PC2 were 53 and 25%, respectively) showed simultaneous presence in samples of DEHP, DIDP, bis-benzilesterephthalate, bis-butilesterephthalate and mono-N-butilesterephthalate, while BPA was related only to the presence of mono-metilesterephthalate (Figure 2). Otherwise, DINP and mono-N-octilesterephthalate were weakly related to others (Figure 2).

Our results are mostly in line with the data present in the literature for other aquatic species, except for DEHP, whose concentration is higher in our samples, especially in the liver. Indeed, DEHP and MEHP concentration were reported in ranges of 9–14.62 ng/g and 1.5–6.30 ng/g, respectively. Instead, according to our data, BPA concentrations in fishes varied from 2 to 75 ng/g in the liver and 1–11 ng/g in the muscle (Belfroid et al., 2002; Fossi et al., 2012; Valton et al., 2014; Guerranti et al., 2016). Probably, the low concentration or absence of contaminants in muscle, gonad and liver could be explained by the strong conversion of these substances to their metabolites after their assimilation (Sackett et al., 2013; Chattopadhyay and Chattopadhyay, 2015). The liver first passage after ingestion convert largely the substances in their metabolites before they reach the other districts. The high concentrations of BPA in gills seems to be related to a major exposure of this substance to the gills’ surface.

These results could be explained by a recent exposure to BPA and, mainly, to phthalates. Since these free substances are largely degraded by marine environment, the high levels of them in fish could be related or to ingestion of microplastics, widely present in marine waters after accumulation and resuspension of plastic debris on the seabed, and the consequent slow releasing of them compounds (Paluselli et al., 2018) or, since these compounds are present in relatively high concentrations in marine water, they can be absorbed through the gills (Barboza et al., 2018).

**FIGURE 3:**
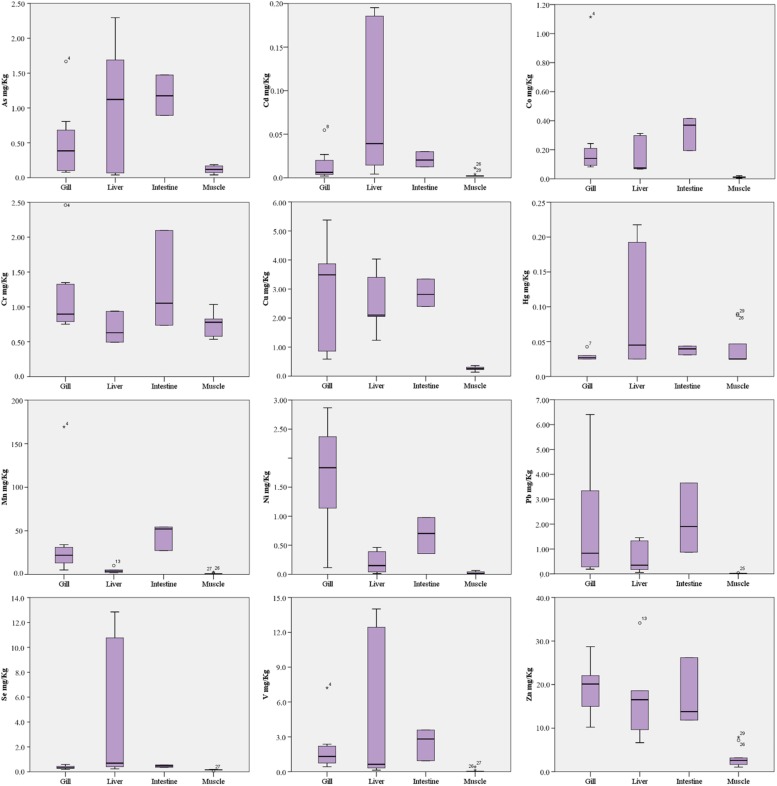
Box plots of trace elements concentrations (mg/Kg ww) in fish tissues.

Trace elements detected in tissue revealed a trend of concentrations generally higher in liver and intestine than in gill and muscle tissues, with the exception of few elements (Table 3 and Figure 3).

**TABLE 3:** Descriptive statistic of trace elements (mg/kg wet weight) in the analyzed tissues.

**Gill**	**As**	**Cd**	**Co**	**Cr**	**Cu**	**Pb**	**Hg**	**Mn**	**Ni**	**Zn**	**Se**	**V**
Min	0.078	< 0.002	0.082	0.752	0.585	0.191	< 0.025	5.058	0.115	10.21	0.195	0.421
Max	1.666	0.055	1.114	2.460	5.375	6.403	0.043	169.3	2.867	28.70	0.592	7.219
Median	0.384	0.006	0.140	0.896	3.490	0.833	0.027	21.92	1.834	20.13	0.321	1.312
Mean	0.510	0.015	0.260	1.154	2.799	1.938	0.029	38.28	1.708	19.16	0.355	2.019
SD	0.533	0.018	0.349	0.578	1.787	2.352	0.006	53.74	0.973	5.693	0.132	2.208
Liver												
Min	0.038	0.004	0.065	0.492	1.236	0.056	< 0.025	1.786	0.011	6.660	0.230	0.131
Max	2.295	0.195	0.312	0.940	4.032	1.456	0.218	10.04	0.462	34.14	12.84	14.01
Median	1.122	0.039	0.074	0.629	3.717	0.353	0.045	3.602	0.149	16.53	0.690	0.633
Mean	1.056	0.080	0.149	0.686	2.488	0.621	0.092	4.377	0.200	17.02	4.271	4.696
SD	0.893	0.088	0.121	0.207	1.027	0.610	0.089	3.142	0.187	9.585	5.875	6.631
Intestine												
Min	0.894	0.013	0.194	0.736	2.400	0.873	0.031	27.25	0.355	11.83	0.355	0.943
Max	1.473	0.030	0.415	2.098	3.346	3.652	0.044	54.39	0.976	26.18	0.556	3.587
Median	1.174	0.020	0.369	1.052	2.813	1.907	0.040	52.07	0.701	13.81	0.468	2.811
Mean	1.180	0.021	0.326	1.295	2.853	2.144	0.038	44.57	0.677	17.27	0.459	2.447
SD	0.259	0.008	0.104	0.637	0.424	1.256	0.006	13.46	0.278	6.954	0.090	1.216
Muscle												
Min	0.039	< 0.002	0.004	0.535	0.137	0.005	< 0.025	0.150	< 0.007	1.056	0.054	< 0.025
Max	0.186	0.011	0.021	1.035	0.360	0.043	0.090	2.445	0.066	7.913	0.189	0.419
Median	0.118	0.002	0.011	0.780	0.257	0.015	0.025	0.487	0.013	2.594	0.153	< 0.025
Mean	0.114	0.003	0.011	0.728	0.257	0.018	0.042	0.722	0.027	3.406	0.147	0.080
SD	0.055	0.003	0.005	0.175	0.065	0.012	0.027	0.768	0.023	2.467	0.039	0.131

In particular, As and Cd were found significantly higher in liver and intestine than gill and muscle tissue (*p* < 0.001 and *p* < 0.05, respectively), although the liver concentrations had a wider value distribution. Co, Cu, Pb, V, and Zn were found with comparable median concentrations in gill, liver and intestine, significantly higher than muscle (*p* < 0.05). Median concentrations of Cr, Hg and Se were comparable in all tissues analyzed, although Hg and Se had a wide value distribution in liver as well as Cr in the intestine. Mn had the highest median concentrations in intestine (*p* < 0.01 vs. other tissues), followed by gill (*p* < 0.01 vs. liver and muscle) and liver (*p* < 0.05 vs. muscle). Ni had the highest median concentrations in gill (*p* < 0.01 vs. other tissues) followed by intestine (*p* < 0.05 vs. liver and muscle).

Several metals represent chemicals known to be used in the plastic industry as additive. Recently, Pb has been phased out as a plastic additive in Europe (Murphy, 2001), while Cd is still under discussion, and is used as pigment, heat and UV-stabilized in PVC. It has been suggested that metals are mostly absorbed onto the pellet surface and are therefore likely bioavailable by ingestion (Ashton et al., 2010). Today the use of some of these metals is restricted, for example As, that is used as antimicrobial and plasticizer (Cordeiro et al., 2012). Thus, contamination of food sources, whether by naturally occurring or introduced toxins, is a concern for consumers due to the adverse health effects that have been associated with exposure to such compounds (Piazzolla et al., 2015; Scanu et al., 2016; Pappalardo et al., 2017; Ferrante et al., 2018a,b).

Few studies in literature report trace element concentrations in *L. caudatus* from the Mediterranean Sea. Naccari et al. (2015) evaluated the presence of Pb, Cd and Hg in specimens collected from the Tyrrhenian and Ionian Seas. They observe the absence of residual levels of toxic metals analyzed in muscle, with concentrations always below the limit of detection, with the exception of Cd and Hg in samples from the Ionian Sea, where they revealed mean concentrations of 0.045 and 0.458 mg/kg ww respectively. Comparable Hg concentrations have been found by Storelli et al. (2007). The authors reported a mean value of 0.59 mg/kg ww in the muscle tissue of *L. caudatus* caught during the summer. These results are higher than concentrations reported in this study (Cd 0.003 mg/kg, Hg 0.042 mg/kg), but they could be influenced by sampling season and by the biological stage of the sampled specimens (Copat et al., 2012). Conversely, Lo Turco et al. (2013) reported metal concentrations in muscle and gills of *L. caudatus*, collected during the winter in the Strait of Messina, which are significantly lower (for all the analyzed metals but not for Cd and As) than concentrations detected in this study, especially in gills. Similarly, to our findings, they found higher metals concentrations in gills respect to muscle tissue, with the exception of As.

**FIGURE 4:**
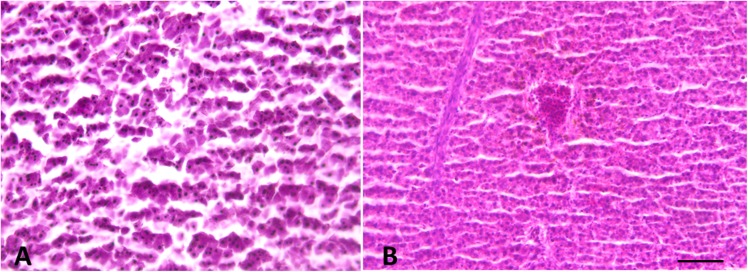
Liver morphology images. **(A)** Male and **(B)** Female. Liver histology showing normal hepatocytes. Scale bar: 200 μm.

**FIGURE 5:**
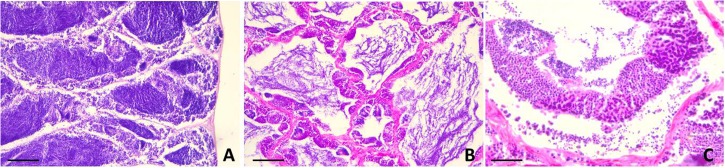
Testis sections of *L. caudatus*. **(A)** anterior portion; **(B)** intermediate portion; **(C)** posterior portion. Sperm cells are visible at different stages of maturation. Scale bar: 200 μm.

**FIGURE 6:**
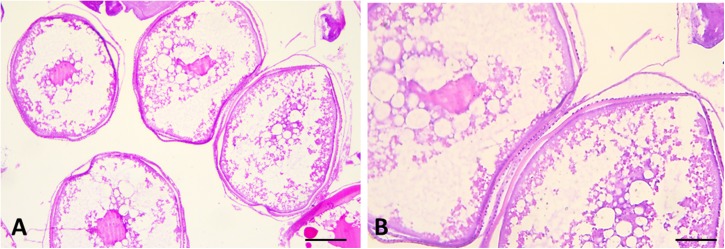
Sections of female gonads of *L. caudatus*. Follicles in different stages of maturation are evident. Scale bar: **A**, 200 μm; **B**, 100 μm.

**FIGURE 7:**
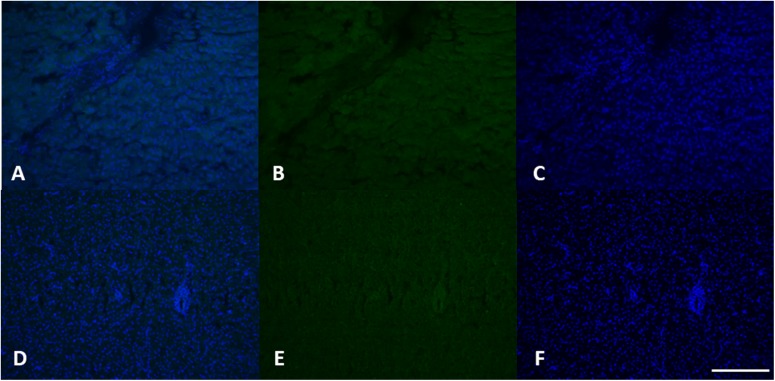
MT 1 expression in female **(A–C)** and male **(D–F)** liver tissue. Using specific antibodies anti-MT1, it has been showed a clear expression of these proteins in the hepatocytes. DAPI staining for nuclei detection (blue). Scale bar: 200 μm2.

Histological analysis did not reveal any morphological alteration of the gills, liver and gonads in both sexes: liver and gonads maintain their typical organization (Figures 4–6); however, immunohistochemical analysis for anti-metallothionein 1 antibody showed a strong positivity of liver cells, both in females (Figures 7A–C) and males (Figures 7D–F) in all 20 the samples analyzed, showing a strong stress that activated a cell detoxification system.

Induction of MTs synthesis, as evidenced with different techniques, is considered a specific and highly sensitive response to heavy metal pollution (Sindermann, 1995; Viarengo et al., 1999, 2000; Cosson, 2000; Amaral et al., 2002). Amaral et al. (2002), for example, found in samples of *Scophthalmus maximus* exposed to different concentrations of cadmium, a significant increase of MTs syntesis in fish liver occurred that was dependent on the cadmium concentration and the exposure time. Authors in fact suggest that MT immunohistochemistry are good tools for clarifying metal and MTs tracking routes in hepatocytes which renders them useful biomarkers of metal exposure.

**FIGURE 8:**
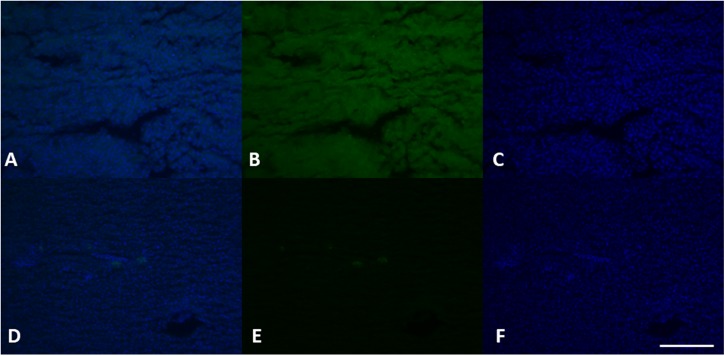
VGT expression in female **(A–C)** and male **(D–F)** liver tissue. Using specific antibodies anti-VGT, it has been showed a clear expression of these proteins in the hepatocytes of female liver, but some positive areas are also evident in some areas of the liver in the male (green). DAPI staining for nuclei detection (blue). Scale bar: 200 μm.

**FIGURE 9:**
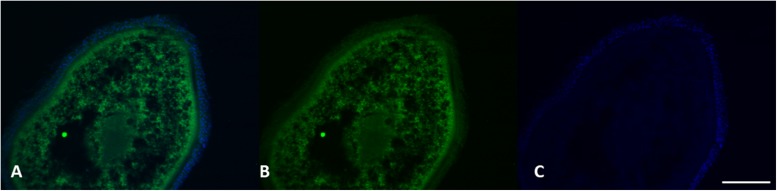
VGT expression in female gonads of *L. caudatus*. It has been showed a clear expression of these proteins (green). DAPI staining for nuclei detection (blue). Scale bar: 200 μm.

**FIGURE 10:**
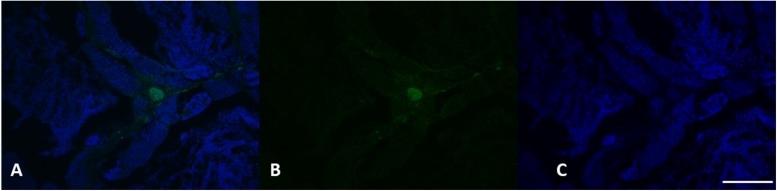
VGT expression in testis of *L. caudatus*. It has been showed a clear expression of this protein in some areas (green). DAPI staining for nuclei detection (blue). Scale bar: 200 μm.

The immunohistochemical analysis for the anti-vitellogenin antibody showed in females a strong positivity both in the liver cells (Figures 8A–C), and in the gonads (Figure 9), as we expected. The analysis of the liver and gonadal preparations of the male specimens was found to be always negative except for a specimen, in which it was highlighted a positivity in some areas of the liver (Figures 8D–F) and in two areas at the level of the gonad in the intermediate portion (Figure 10). These results show that the specimen has been exposed to contaminants that can act as endocrine disruptors, activating the gene expression for vitellogenin, which in males and immature females is normally silenced (Matozzo et al., 2008).

The presence of plastic additives and heavy metals could be related to ingestion, accumulation and low degradation of microplastics as it has been documented in marine organisms belonging to different species and with different feeding habits. In fact, due to their small size, microplastics can be accidentally ingested during filtration or ingestion of prey. In particular, plastic particles have been found in more than 100 marine species, from zooplankton to whales, including marine reptiles and birds (Gallo et al., 2018). Marine organisms can ingest microplastics by mistaking them for prey (Cole et al., 2011; Campani et al., 2013; Wright et al., 2013; Romeo et al., 2016) or accidentally during feeding (Fossi et al., 2014). Once intaked, the microplastics accumulate in the intestine, move into other tissues or are expelled, depending on the size, shape and composition (Gallo et al., 2018). In sea crab (*Carcinus maenas*) and in several filtering molluscs, it has been shown that not only they ingest the microplastics together with food, but these particles can remain entrapped in gills (Moore, 2008; Watts et al., 2014). Goldstein and Goodwin (2013) have assessed the potential effects of microplastic on the rafting community, examining the gastrointestinal tracts of 385 barnacles collected from the North Pacific Subtropical Gyre (NPSG) for evidence of plastic ingestion. They found that 33.5% of the barnacles had plastic particles present in their gastrointestinal tract. Their results suggest that barnacle ingestion of microplastic is relatively common, but with unknown trophic impacts on the rafting community and the NPSG ecosystem.

Ingestion of microplastics has been verified also in the stomach of the Humboldt squid (*Dosidicus gigas*) (Braid et al., 2012), a large predator that usually feeds at depths between 200 and 700 m, although the ingestion route is not yet clear.

Plastic fibers, probably derived from fibers of trawl nets and fragments of plastic bags, have been identified in the intestine of the Norway lobster, *Nephrops norvegicus* (Murray and Cowie, 2011): these organisms have various modes of feeding, including scavenging and predation, but they are not suitable for cutting flexible filamentary materials that are not eliminated by the normal digestive processes.

The results obtained confirm that the exposure to plastics additives of marine organisms is a widespread phenomenon and underlines the environmental relevance of the problem of plastic waste at sea. It is therefore urgent that scientific research acquires new knowledge and helps to raise awareness of this emerging issue.

Although the presence of microplastics in the intestine of the studied species could not be confirmed, the results obtained suggest an exposure of marine organisms to plastics additives (phthalates, bisphenol A and heavy metals), thus underlining the environmental relevance of the problem of plastic waste in the sea. It is therefore urgent that scientific research acquires new knowledge and helps to raise awareness of this emerging issue

## Conclusion

The existence of microplastics and their potential impact on wildlife has received more public and scientific attention in recent years (Betts, 2008; Galloway, 2015; Lusher, 2015). Indeed, it is only with the recent European (Marine Strategy Directive Marine Strategy Directive [MSFD, 2008/56/EC], 2008) that the microplastics have been inserted among the contaminants worth to be monitored. However, at the moment, any analytical method is reliable to investigate microplastics, in particular those having diameters smaller than 10 μm. In fact, these fragments, due to their ability to be adsorbed by digestive and respiratory systems and, consequently, to be largely distributed in all part of body, are the most dangerous for health (Powell et al., 2010; Wright and Kelly, 2017).

This study confirmed the presence and the potential endocrine disruption effect of chemicals used as and/or associated with plastic additives in *L. caudatus*, thus suggesting the potential bioaccumulation of microplastics in marine organisms and the serious threat for aquatic systems and human health. The results obtained provide a further contribution to the knowledge on the impact of plastic wastes and especially of associated contaminants on marine life and on fish species of commercial interest. This study also confirms the validity of the triple monitoring approach proposed by Fossi et al. (2018), and the role of vitellogenin as a valid biomaker of exposure to endocrine disruptors.

## Author Contributions

AS, FT, and MB developed the research idea and experimental design. FT, DM, and GM collected the specimens. AM, RP, and ES made histological and immunohistochemical analysis. MB, CC, and PZ co-wrote the manuscript. EK, IG, RC, BL, and MF reviewed the manuscript. All authors read and approved the final manuscript.

## Conflict of Interest Statement

The authors declare that the research was conducted in the absence of any commercial or financial relationships that could be construed as a potential conflict of interest. The reviewer AZ and handling Editor declared their shared affiliation at the time of review.
